# Degradation pathway of triazole fungicides and synchronous removal of transformation products via photo-electrocatalytic oxidation tandem MoS_2_ adsorption

**DOI:** 10.1007/s11356-020-12185-x

**Published:** 2021-01-02

**Authors:** Junwen Wang, Xiaoxin Chen, Xiaoli Sun, Miao Liu, Xingqiang Wu, Yichao Gong, Jianfang Du

**Affiliations:** 1 College of Chemistry and Environmental Science, Hebei University, Baoding City, 071002 Hebei Province China; 2 Key Laboratory of Mineral Resources and Eco-environment Monitoring, Hebei Province, Baoding, China; 3 Bioengineering Technology Innovation Center of Hebei Province, Baoding, China

**Keywords:** Photo-electrocatalytic oxidation, Degradation pathway, Triazole fungicides, Synchronous removal, Tandem process

## Abstract

A simple and effective tandem process of photo-electrocatalytic oxidation (PECO)-MoS_2_ adsorption was developed for the synchronous removal of triazole fungicides (TFs) and toxicological transformation products (TPs). In order to accurately identify trace TPs and evaluate degradation pathway during water treatment, a sensitive analytical method was developed on the basis of the stir bar sorptive extraction (SBSE) pretreatment tandem LC-MS/MS technique. Firstly, the typical TFs (PRO, TET, and DIN, *C*_0_ = 1.0 mg/L) in actual water samples were treated under the optimal process (bias voltage 1.8 V, pH 4, irradiation intensity 50 mW/cm^2^, 0.05 g MoS_2_/100 mL, 350 rpm, adsorption of 5 min). The result indicated that the residues of PRO, TET, and DIN in secondary effluent were 0.0973, 0.0617, and 0.0012 mg/L, respectively, with the removal rates of 90.3%, 93.8%, and 99.9%, respectively, undergoing 30-min photo-electrocatalysis and 5-min adsorption. The alkaline medium was favorable for the adsorption of MoS_2_ to TFs. The assessment results of potential cancer risk indicated that the residues of TFs in secondary effluent were safe for drinking water consumption. Besides, the major TPs were identified via the SBSE-HRLC-MS/MS technique, and one possible transformation pathway of TFs was proposed. TFs mainly underwent dehydrochlorination, cyclization, hydroxylation, etc. to produce a series of nitrogenous heterocyclic compounds that possess higher polarity than parents, hinting that TPs might pose potential aquatic toxicity. However, TPs can be removed synchronously by this tandem technique. The current study can provide a theoretical basis for the harmless treatment of TFs in the water environment.

**Supplementary Information:**

The online version contains supplementary material available at 10.1007/s11356-020-12185-x.

## Introduction

In order to increase the yield and harvest of agricultural products, triazole fungicides (TFs) have been widely used in agricultural production due to the characteristics of low toxicity, high efficiency, and broad spectrum. However, TFs cause different degrees of pollution to rivers, lakes, and groundwater around the world (Climent et al. 2019; Ccanccapa et al. 2016). For example, the average residue of propiconazole in Tengger River Basin in Malaysia is as far as 4493.1 ng/L (Elfikrie et al. 2020) and approximately 0.291–1.150 μg/L in the soybean-growing agricultural surface water in south central United States (Battaglin et al. 2011). The residue of tebuconazole in European rivers reaches the level of 175–200 μg/L (Wang et al. 2011). More and more in vitro and in vivo experiments show that TFs exhibit hepatotoxicity (Zhang et al. 2019), reproductive development toxicity (Tian et al. 2019), and endocrine disruption (Draskau et al. 2019) to non-target organisms. TFs have been listed as one of the “potential carcinogens of human beings” by the U.S. Environmental Protection Agency (USEPA) (Crowell et al. 2011). Although the residues of TFs in the water environment are at the trace level (ng/L or μg/L), some TFs such as propiconazole, tebuconazole, and prothioconazole still cause teratogenesis, carcinogenesis, and mutation (Tian et al. 2019; Teng et al. 2019). Therefore, even trace TFs pose a potential threat to the water environment and human health. On the other hand, pesticides are a kind of typical environmental exogenous substances; once they enter into the environment, they will be degraded or metabolized to a series of unknown transformation products (TPs) under the biologic and abiotic actions. Numerous studies indicated that TPs often show stronger polarity, higher persistence, higher bioaccumulation factors, and diverse action pattern than parent itself. It is reported that 51% of TPs are equally or more toxic than parents, in which the toxicity of 9% TPs is more than 10 times (Boxall et al. 2004). It is reported that TFs are usually transformed into a series of nitrogenous heterocyclic compounds (NHCs) with unknown chemical properties in the existence of light exposure (Rodríguez-Cabo et al. 2018). However, these existing technologies mainly focus on the degradation of pesticide parent, but little attention is paid to their toxicological TPs (Ying and Luo 2017; Cui et al. 2017), which will inevitably lead to secondary pollution. In view of this, how to achieve synchronous removal and detoxification of TFs and trace toxicological TPs in the water environment becomes an important problem to be solved urgently at present.

As far as we know, the existing degradation technologies of TFs mainly include biochemical process (Wu et al. 2018; Wu et al. 2019), adsorption (Masis-Mora et al. 2019), advanced oxidation processes (AOPs) such as Fenton oxidation (Zhang et al. 2020), photo-catalytic oxidation (Garcia-Muñoz et al. 2020), and electrocatalysis (Cui et al. 2017). At present, the AOPs have been developed rapidly in degradation of organic pollutants, because of strong oxidation ability, mild reaction conditions, fast reaction rate, and good treatment effect (Zhang et al. 2020; Garcia-Muñoz et al. 2020). Among various AOPs, the photo-electrocatalytic oxidation (PECO) technique fixes the catalyst on a conductive substrate, and the external bias can effectively promote the separation of photo-generated electrons and holes, thus improving the quantum efficiency significantly. Owing to the increase of ·OH on semiconductor surface, PECO exhibits higher catalytic efficiency in degrading organic pollutants than single photo-catalysis or electrocatalysis (Wang et al. 2017). Although the PECO technique is described in many papers in literatures, the research on photo-electrocatalytic degradation of TFs is very scarce.

Based on the dispersion state of catalyst, the photo-electrocatalytic reactor (PECR) often includes slurry reactor (Molinari et al. 2017) and immobilized reactor (Jaramillo-Gutiérrez et al. 2020); therein, the former has some shortcomings, such as a complex separation process of the catalyst and lower reusability (Christensen et al. 2003), while the latter can avoid these problems. At present, the most commonly used immobilized PECR is the cylindrical reactor equipped with a ring electrode, which can avoid light energy attenuation due to the distance reduction between the light source and photo-anode (Zhao et al. 2014). In addition, mesh electrode is beneficial to enhance mass transfer. Besides, the photo-electrocatalytic efficiency of the tubular reactor can be enhanced by aeration (Suhadolnik et al. 2019). However, the computational fluid dynamics (CFD) showed that there are non-uniform flow and dead volume at the inlet and outlet of the closed tubular reactor (approximately 35% of the total reactor volume) (Matos et al. 2018). Therefore, we developed a bench-scale submerged reactor with annular network anode on the basis of tubular PECR. Firstly, the designed reactor was in an open state, in which it does not require additional aeration. Secondly, the submerged reactor itself was used as a liquid storage tank that can achieve degradation reaction and water sample storage at the same time, thus reducing the dead volume partly. Besides, the combination of a magnetic stirrer and a circulating pump strengthened the mass transfer.

In order to better understand and guide the harmless treatment of TFs, it is necessary to pay more attention about degradation behavior and transformation pathway when developing a novel treatment technique, because a clear degradation pathway will facilitate the feedback regulation and optimization of process parameters, thus achieving the final goal of green resistance and ecological remediation. However, in order to identify target pollutants accurately and elucidate degradation pathway, there put forward higher requirements about analytical methods because of trace unknown-property TPs. Hence, it is necessary to construct a precise identification system that provides accurate information to reflect the degradation behavior and transformation pathway of TFs. As is universally known, the high-resolution chromatography tandem mass spectrometry (HRLC-MS/MS) technique can provide an important support for the accurate quantification of trace compounds in a complex matrix, as well as the structural identification of unknown compounds. Besides, the stir bar sorptive extraction (SBSE) is a simple and efficient pretreatment method with good reproducibility and sensitivity, and there only needs a little liquid samples and organic solvents to extract and enrich ultra-trace compounds from the complex matrix (Aparicio et al. 2017; Ochiai et al. 2013). The combination of SBSE pretreatment and the selected ion detection mode of LC-MS/MS can ensure the limit of detection (LOD) of ng/L level (Grau et al. 2019).

The major objectives of this study are as follows: (1) to construct a bench-scale submerged PECR using IrO_2_/RuO_2_/TiO_2_/Ti-based photosensitive mesh electrodes; (2) to clarify the degradation behavior and transformation pathway of TFs at the molecular level on the basis of the SBSE-HRLC-MS/MS technique; (3) to evaluate the practicability of the PECO-MoS_2_ adsorption technique in the synchronous removal of TFs and their TPs, as well as the potential cancer risk of TFs in effluent to the human body. This study has an important theoretical value and practical significance for green resistance control and ecological restoration of TFs in the water environment.

## Materials and methods

### Chemicals and instruments

The standards of diniconazole (99.0%) and propiconazole (99.0%) were purchased from Beijing Qincheng Yixin Technology Development Co., Ltd. (Beijing, China). Tetraconazole standard (98.8%), HPLC-grade acetonitrile, methanol, and formic acid were purchased from J&K Scientific Ltd. (Beijing, China). The analytical-grade reagents in the experiments were obtained from Aladdin Reagent Co., Ltd. (Shanghai, China). Ultra-pure water was supplied by a local supermarket. MoS_2_ and WS_2_ nanoparticles were obtained from Guangdong Mitak Metal Materials Co., Ltd. (Guangzhou, China). The Nylon microfilter (0.22 μm) was provided by Peak Sharp Company, P. R. China. The bipolar ACAR/PDMS stir bar (ZZ-SBSE-2-01, 20 mm length × 1.0 mm thickness) was provided by Qingdao Zhenzheng Analytical Instrument Co., Ltd. (Qingdao, China). The acetic acid-sodium acetate buffer solution (pH 4.0) was prepared with 3.6 mL sodium acetate solution (1.0 mol/L) and 16.4 mL acetic acid solution (1.0 mol/L). Standard stock solutions of individual diniconazole (5000 mg/L), propiconazole (9830 mg/L), and tetraconazole (1976 mg/L) were prepared using HPLC-grade acetonitrile in a brown volumetric flask, respectively. The standard solutions were stored at − 20 °C to maintain stability within 1 year. The intermediate standard mixture solution (50 mg/L) of propiconazole (PRO), tetraconazole (TET), and diniconazole (DIN) was prepared by diluting stock solutions with HPLC-grade acetonitrile. The individual working aqueous solutions (1.0 mg/L) of PRO, TET, and DIN and the mixed working solutions (1.0 mg/L) of three pesticides were prepared with deionized water, respectively, according to the following method. An appropriate amount stock solution was pipetted and transformed into a 100-mL flask, and solvent evaporation was performed by an evaporator under 40 °C water bath. Finally, the target pesticides were reconstituted with 2.0 L deionized water by ultrasound using ethanol (0.05%, v:v) as a cosolvent. IrO_2_/RuO_2_/TiO_2_/Ti-based photosensitive coating mesh electrodes (cylindrical anode 24 cm length × 4 cm i.d., rhombus mesh density of 4 cm^−2^; rectangular sheet cathode 24 cm length × 4 cm width, rhombus mesh density of 4 cm^−2^) were customized by Mingxuan Titanium Products Co., Ltd (Xingtai, Hebei). The tank of bench-scale submerged PECR (28 cm length × 12 cm width × 10 cm height, thickness 1.0 cm, side hole diameter 35 mm, 2.5 L) was provided by Shanghai Acrylic Co., Ltd. (Shanghai, China). A rotary evaporator (RV 10 digital, IKA (Guangzhou) Instrument Equipment Co., Ltd.) was used to evaporate solvent. For the quantification of TFs and identification of TPs in aqueous solution, a high-performance liquid chromatography (LC-20AT, Shimadzu) tandem triple quadrupole mass spectrometer (ABS Triple Quad 5500, USA) (HPLC-QqQ-MS/MS) equipped with a reversed-phase C18 column (100 mm × 4.6 I.D., 5 μm, Kromat Corporation) and a Dual JetSpray electrospray ionization source in the positive ion mode (ESI^+^) was employed in this study.

### Stir bar sorptive extraction pretreatment

To enrich the trace TPs, the SBSE pretreatment method was used in this study as the following procedure. The ACAR/PDMS stir bar was preconditioned with 1.0 mL methanol by ultrasound for 20 min and dried at 70 °C. The liquid sample (2.0 mL) was transferred to a 50-mL glass beaker. Subsequently, the pH was adjusted to 4 with acetic acid-sodium acetate buffer solution. After the preactivated stir bar was put into the buffer solution system, the solution was stirred magnetically at 600 rpm for 40 min, to extract target pollutants from water samples. Afterwards, the stir bar was placed into another extraction flask adding into 500 μL methanol-acetonitrile mixture (1:1, v:v), and the target analytes were desorbed for 20 min by ultrasound. Finally, the extract was filtered through a 0.22-μm fiber prior to LC-MS/MS analysis. Three parallel treatments were performed for each.

### PECR construction

A well-designed reactor can increase the degradation rates of TFs. In this study, a bench-scale PECR (280 mm length, 12 mm width, 10 mm depth, plexiglass material) was designed and constructed as shown in Fig. S1. An ultraviolet lamp radiating 254 nm wavelength was matched with ballast and quartz lamp sleeve, and 30 mW/cm^2^ and 50 mW/cm^2^ was set for degradation of TFs, respectively. The lamp device was used as the inner light source of the reactor in immersion mode. The mesh cylinder anode was covered outside the light source, while the mesh sheet cathode was placed vertically in the solution. The anode and cathode were connected to the direct-current power. The reactor was placed onto a multichannel magnetic stirrer (SP200-2T, Hangzhou Miou Instrument Co., Ltd, China). Meanwhile, a small circulating pump (flow rate of 2.0 L/min) was connected to the reactor with a silica gel tube to enhance mass transfer.

### Photo-electrocatalytic degradation of TFs

#### Parameter optimization

The Na_2_SO_4_ was used as the electrolyte in the process of photo-electrocatalysis. The aqueous solution of TFs (*C*_0_ = 1.0 mg/L, 0.05 mol/L Na_2_SO_4_, 2.0 L) was investigated under different operation parameters of reactor. The effects of bias voltage (1.8, 2.0, 2.5, and 3.0 V), irradiation intensity (30 mW/cm^2^ or 50 mW/cm^2^), initial concentration (*C*_0_ = 1.0, 5.0, and 20 mg/L), pH (4, 7, and 9), and iron ion (Fe(II), 0–2.0 mg/L) on degradation rates of were investigated. The ferrous sulfate Fe(II) with different amounts was added into the mixture (*C*_0_ = 1.0 mg/L) of PRO, TET, and DIN. The pH values of aqueous solutions were adjusted to 4, 7, or 9 with dilute sulfuric acid or sodium hydroxide solution. Sampling (2.0 mL) was performed at the intervals and pretreated according to the above procedure in “Stir bar sorptive extraction (SBSE) pre-treatment.” Finally, the residues of target compounds were analyzed in the multiple reaction monitoring (MRM) mode of the HPLC-MS/MS technique after filtered by a 0.22-μm filter membrane. The mobile phase was the mixture of acetonitrile (A) and 0.2% formic acid aqueous solution (B) (80:20, v:v) with the flow rate of 0.2 mL/min. The column temperature was controlled at 30 °C. The injection volume was 1.0 μL. The mass spectrometry parameters were as follows. The ion source temperature was controlled at 200 °C. The flow rate and pressure of nebulizer gas (N_2_, 450 °C) were 11 L/min and 40 psi, respectively. The capillary voltage was 4000 V. The fragment voltage was 120 V for TET and 100 V for both PRO and DIN, respectively. The selected quantitative (qualitative) ion pairs for PRO were 342.1/159.1 (342.1/70) and 372/159 (372/185) for TET and 326.1/70 (326.1/158.7) for DIN. The mass data acquisition was performed by the Multi Quant Workstation Software (version 3.0).

#### TP identification

The individual aqueous solutions of PRO, TET, and DIN (*C*_0_ = 1.0 mg/L, 0.05 mol/L Na_2_SO_4_, 2.0 L) were degraded for 30, 30, and 15 min, respectively. Samples (2.0 mL) were collected and pretreated according to the procedure in “Stir bar sorptive extraction (SBSE) pre-treatment.” These TPs were analyzed in a scan mode of HPLC-MS/MS with the range of *m*/*z* 100–450, and the PI mode was used to obtain adequate mass spectrometry data from a mass analyzer. The mobile phase was the mixture of acetonitrile (A) and 0.2% formic acid aqueous solution (B) with the volume ratio of 60:40 (PRO), 60:40 (TET), and 50:50 (DIN). The flow rate was 0.4 mL/min. The stop time was 30 min. The column temperature was controlled at 30 °C. The injection volume was 20 μL for PRO (or TET) and 25 μL for DIN. The ion source temperature was controlled at 450 °C. The ESI^+^ was operated. The column temperature was controlled at 35 °C. The 40-psi N_2_ was used as a nebulizer. The flow rates for drying gas (325 °C) and sheath gas (325 °C) were 10 L/min and 11 L/min, respectively. The capillary voltage was controlled at 4000 V.

### Adsorption of TFs and TPs by nano-MoS_2_

#### Optimization of adsorption conditions

To obtain optimum adsorption effects of TF parent and their TPs, the PRO aqueous solution (*C*_0_ = 1.0 mg/L) was firstly degraded for 60 min by the reactor device. Subsequently, the primary effluent was used to optimize the adsorption process. The major operation parameters including dosages of MoS_2_ or WS_2_, initial concentration (*C*_0_), pH, adsorption temperature, and stirring speed were optimized in this study. The dark control was performed under the light-free condition with tinfoil wrapping. The samples were collected at the intervals of 0, 1, 2, 5, 10, 15, 20, and 30 min and pretreated according to the SBSE pretreatment method. The residues of PRO and their TPs were analyzed via the HPLC-MS/MS technique after filtered by a 0.22-μm filter as described in “Stir bar sorptive extraction (SBSE) pre-treatment.” In order to verify the actual removal effect of optimum adsorption unit to TFs and their TPs, the mixed aqueous solution of PRO, TET, and DIN (*C*_0_ = 1.0 mg/L) was firstly degraded for 30 min. Subsequently, the target pollutants in primary effluent were adsorbed under the optimum adsorption conditions.

#### Adsorption capacity of MoS_2_

The adsorption effects of MoS_2_ to TFs can be estimated via separation factor (*R*_L_) because it is related to the surface activation energy of adsorbent. The adsorption will be easy under experimental conditions when *R*_L_ is in the range of 0–1. The lower *R*_L_ facilitates the adsorption of MoS_2_ to TFs. Firstly, the Langmuir adsorption isotherm equation was plotted by the regression analysis of *C*/*Q* versus *C* according to Formula (2). Subsequently, *R*_L_ was calculated according to Formula (3).

1 $$ Q=\frac{\left({C}_0-C\right)\times V}{m} $$

2 $$ \frac{C}{Q}=\frac{1}{Q_{\mathrm{e}}}\times C+\frac{1}{Q_e\times b} $$

3 $$ {R}_L=\frac{1}{1+b\times {C}_0} $$

where *C*_0_ and *C* are the initial and equilibrium concentration (mg/L) of pollutants in water phase, respectively. The *m* was the dosage of MoS_2_. The variable *V* was the volume (L) of water sample. *Q* and *Q*_e_ were the isotherm adsorption capacity (mg/g) and the saturated adsorption capacity (mg/g), respectively. The parameter *b* was the adsorption equilibrium constant.

### Practicability of the PECO-MoS_2_ adsorption technique

The actual surface water was firstly pretreated by a 0.45-μm water microfilter to remove particulate impurities. Then, the filtrate was spiked with the standard mixture solution of PRO, TET, and DIN to obtain the simulated water samples (1.0 mg/L). Firstly, the water samples were treated for 30 min via the established reactor. Subsequently, the primary effluent (100 mL) was treated under the optimum adsorption process to remove TF parents and their TPs synchronously. The degradation rate of target pollutants was evaluated on the basis of dynamic data. Meanwhile, the total organic carbon (TOC) was detected by a TOC analyzer. These samples were pretreated in triplicate for each.

### Carcinogenic risk assessment of effluent

It is necessary to predict the potential cancer risk of effluent to the human body, because it will further guide the process improvement. In this study, the carcinogenic risk coefficient (*P*) of individual TFs in effluent was evaluated on the basis of the residual concentration (*C*). The adult average weight (65 kg) and daily water intake (2.0 L/day) were used for risk assessment as the following formula.

$$ P=\frac{C\left(\mathrm{mg}/\mathrm{L}\right)\times {10}^{-6}\times 2.0\mathrm{L}}{65\mathrm{kg}}\times f $$

According to USEPA-2008, the control standard of carcinogenic risk induced by drinking water containing chemical pollutants should be in the range of 10^−6^–10^−4^. In view of the maximum risk principle, 10^−6^ was referenced as the safe drinking standard in this study. Moreover, 1.0 was considered the carcinogenic potency factor (*f*) of target analyte in order to improve the process based on the most stringent standard.

## Results and discussions

### Photo-electrocatalysis kinetics of typical TFs

**Fig. 1:**
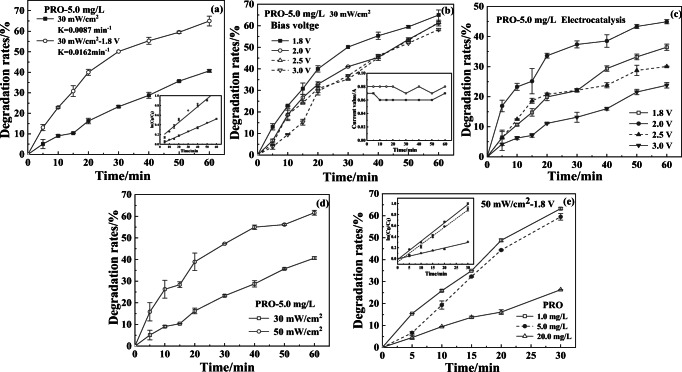
Effects of bias voltage (**a**, **b**), electrocatalytic voltage (**c**), light intensity (**d**), and initial concentration (**e**) on degradation rates of PRO in aqueous solution (0.05 mol/L Na_2_SO_4_)

The effects of bias voltage, initial concentration (*C*_0_), irradiation intensity, pH values, and Fe(II) on degradation rates of PRO, TET, and DIN were investigated. As shown in Fig. 1a, the rate constants of photo-electrocatalysis and photo-degradation of PRO were 0.0162 min^−1^ (*R*^2^ = 0.9861) and 0.0087 min^−1^ (*R*^2^ = 0.9982), respectively, indicating that the photo-electrocatalytic efficiency was higher than that of single photo-degradation under the same conditions. As shown in Fig. 1b, a downward trend about the degradation rates of PRO was observed with the increase of bias voltage (1.8–3.0 V). This result was mainly attributed to the competitive oxygen evolution reaction (OER) induced by water electrolysis, because some bubbles were escaped from the anode surfaces, and there were more and more bubbles with the increasing bias voltage (> 2.0 V). This viewpoint was supported by the published literature (Li et al. 2000). Although the increase of bias voltage was conducive to the degradation of organic pollutants to a certain extent, the higher bias voltage (beyond oxygen evolution potential) not only makes water electrolysis the main reaction on the electrode, but also causes serious anode pollution (Rodgers and Bunce 2001). We found that the electrocatalytic removal rate of PRO began to decline when the voltage exceeded 2.0 V (Fig. 1c). This result further confirmed that the applied bias voltage should be below 2.0 V under the experimental conditions in this study. On the other hand, Fig. 1b indicates that the electric current was basically unchanged with degradation time under the constant voltage (1.8 V or 2.0 V). This result suggested that the number and rate of photo-generated electrons moving to the opposite electrode remain unchanged during the photo-electrocatalytic degradation process. In other words, the photo-electrocatalytic effect was basically unchanged under the bias voltage of 1.8 V or 2.0 V. Furthermore, the degradation rates of PRO (*C*_0_ = 5.0 mg/L, 0.05 mol/L Na_2_SO_4_) were 65.0% (1.8 V) and 61.3% (2.0 V), respectively, under 60-min photo-electrocatalysis. Considering the above-mentioned reasons comprehensively, 1.8 V was applied to the photosensitive electrodes for the degradation of TFs. Figure 1d indicates that the higher irradiation intensity contributed to the degradation of pollutants. As seen from Fig. 1e, the photo-electrocatalytic degradation of PRO was well in line with the first-order kinetics (*R*^2^ > 0.9938). The rate constants were 0.0334 min^−1^ (1.0 mg/L), 0.0315 min^−1^ (5.0 mg/L), and 0.0099 min^−1^ (20 mg/L), respectively. The lower rate constant at a lower initial concentration might be mainly depended on the number of active sites on electrode surface. However, it is worth noting that although the decomposition was faster at lower concentrations, the removal amounts of TFs were higher at greater concentrations.

The effects of pH and Fe(II) on degradation kinetics of DIN, TET, and PRO in aqueous solution are described in Fig. S2(a–f). Fig. S2(a) indicated that DIN was easily degraded in acid, neutral, or alkaline media without meeting the first-order kinetics, and the degradation rates of DIN (*C*_0_ = 1.0 mg/L) within 5 min were up to 95.84% (pH = 4), 91.01% (pH = 7), and 94.38% (pH = 9), respectively. Almost no degradation of DIN was observed after 5 min. However, the photo-electrocatalysis degradation of PRO and TET in aqueous solutions followed pseudo-first-order kinetics remarkably, with satisfactory linear relations of ln(*C*_t_/*C*_0_) versus time (*t*) (*R*^2^, 0.9768–0.9930) (Fig. S2(b, c)). It was easily observed from the curve slopes that the degradation rates of both PRO and TET in acidic medium were higher than that of alkaline. As a kind of weak acid pesticide, TFs in acid medium mainly existed in the molecular form, in which it was easily adsorbed on the electrode surface; thus, photo-electrocatalysis was favorable. Besides, the theoretical redox potentials of ·OH/H_2_O based on the Nernst equation were 2.354 V (pH 4), 2.177 V (pH 7), and 2.059 V (pH 9), respectively (Table S1). These data also showed that acidic medium was beneficial to the degradation of TFs (Mazierski et al. 2019). An accelerating effect as to the degradation of PRO and TET was observed with the presence of Fe(II) in the range of 0.1–2.0 mg/L (Fig. S2(e, f)), because Fe(II) mainly exists in aqueous solution in the dissolved forms of Fe^2+^, Fe^2+^, FeOH^2+^, and Fe_2_(OH)_2_^4+^, in which FeOH^2+^ could be photo-active to the decay of PRO and TET due to the photo-generated hydroxyl radicals (Chen et al. 2016). However, this photosensitization was almost not reflected in DIN degradation (Fig. S2(d)), which might be due to the rapid degradation rate.

### Transformation pathway of TFs

**Fig. 2:**
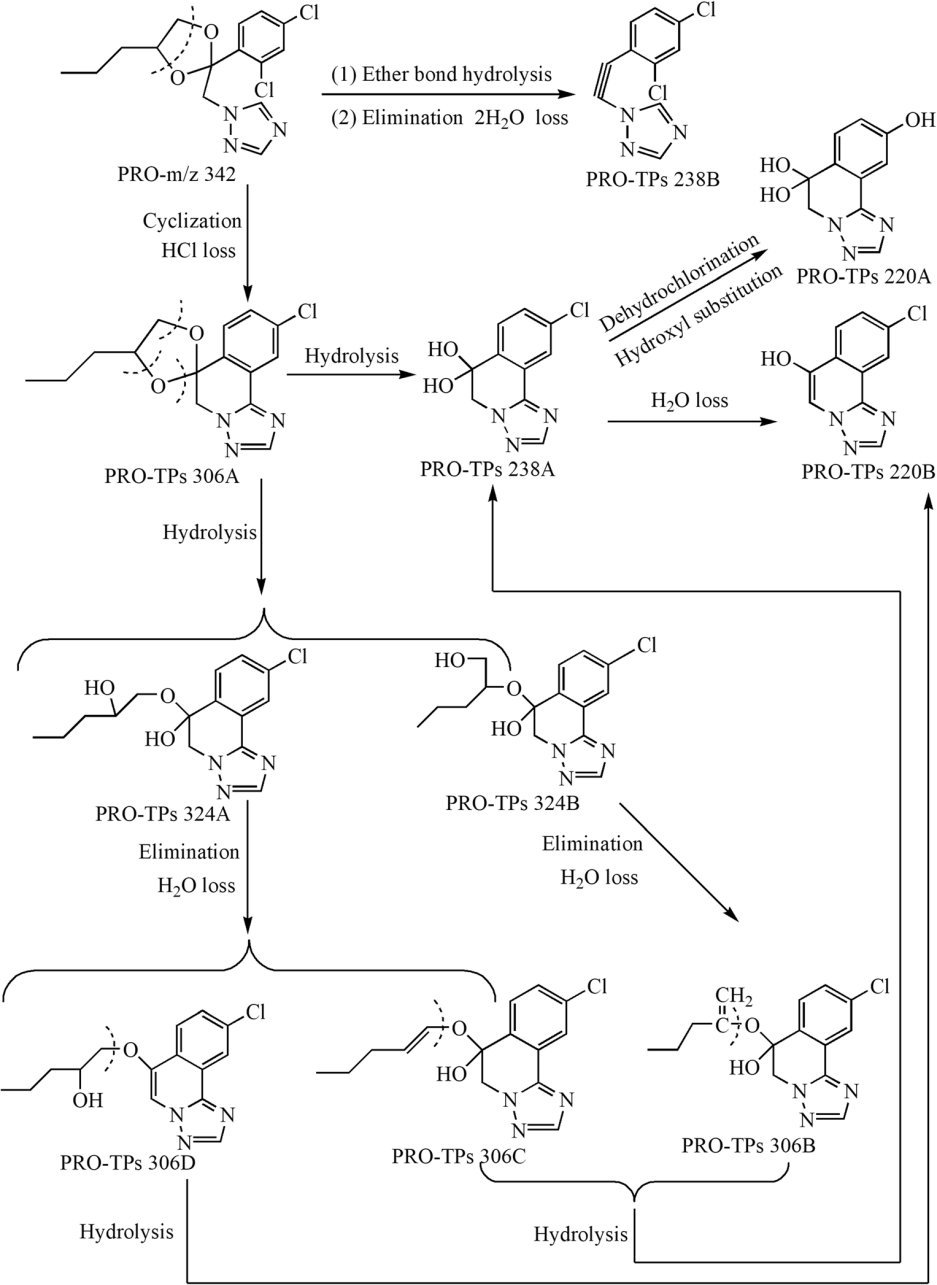
TPs of PRO in aqueous solution and transformation pathway

**Fig. 3:**
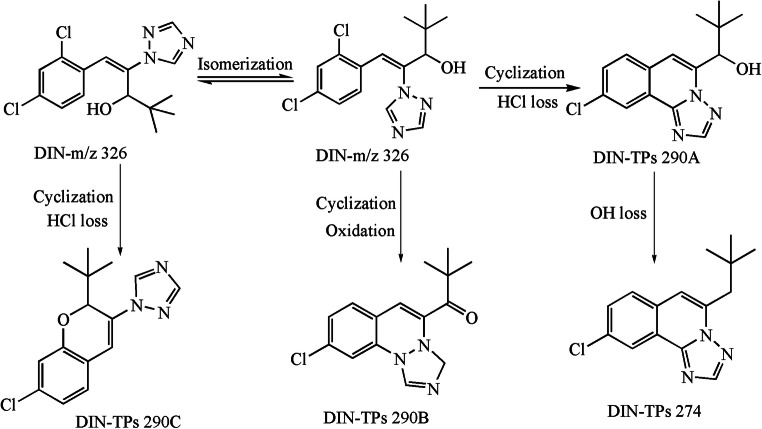
TPs of DIN in aqueous solution and transformation pathway

**Fig. 4:**
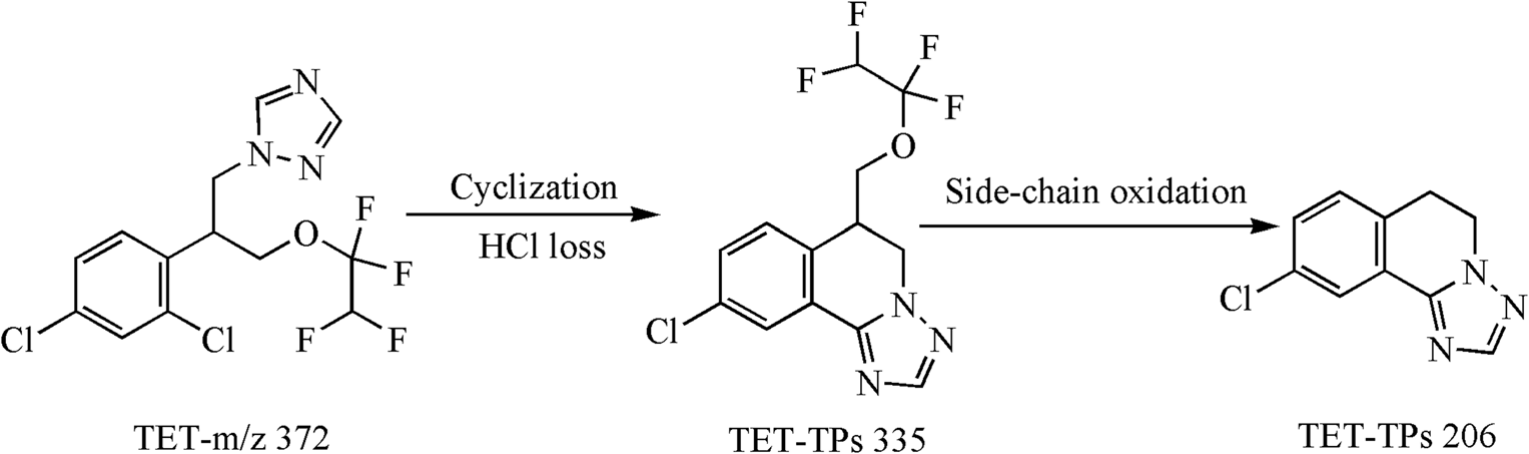
TPs of TET in aqueous solution and transformation pathway

The major TPs of three typical TFs (PRO, TET, and DIN) were identified on the basis of the accurate MS/MS data (their protonated molecules, MS/MS fragmentation patterns, and reasonable fragment loss regulations) (Tables S2–S4 and Figs. 2, 3, and 4). As shown in Table S2 and **Fig.** 2, there generated ten byproducts with PRO photo-electrocatalysis degradation. PRO-TPs306A was derived from cyclized dehydrochlorination of PRO parent, and it included the characteristic fragments of 238 and 220 corresponding to the loss of C_5_H_8_ and (C_5_H_8_+H_2_O), respectively. The two ether bonds of PRO-TPs306A were further hydrolyzed to form PRO-TPs238A with two H_2_O losses. However, PRO-TPs238B was generated from the ether bond hydrolysis of the PRO parent along with two H_2_O eliminations. Although PRO-TPs238A and PRO-TPs238B exhibited the same characteristic fragment of *m*/*z* 220, they represented the loss of H_2_O molecule and hydroxylation of chlorine atom, respectively. Owing to higher polarity of two hydroxyl groups, PRO-TPs238A (*R*_t_ = 2.0 min) was eluted preferably in the C18 reversed-phase column than PRO-TPs238B (*R*_t_ = 4.0 min). The substitution of chlorine atom by the hydroxyl group on the benzene ring of PRO-TPs238A caused the formation of PRO-TPs220A, while underwent the elimination of H_2_O molecule to form PRO-TPs220B. The reasonable fragmentation loss patterns were beneficial for verifying the molecular structure of PRO-TPs220A and PRO-TPs220B (Table S2). Both PRO-TPs324A and PRO-TPs324B came from the hydrolysis of ether bond of PRO-TPs306A. PRO-TPs324A showed the fragments of *m*/*z* 306, 283, 265, 255, and 238, representing the loss of H_2_O, (C_3_H_5_), (C_3_H_5_ + H_2_O), (C_3_H_5_ + CO), and (C_3_H_5_ + H_2_O + C_2_H_3_), respectively. The characteristic ions (*m*/*z* 306, 238, and 220) of PRO-TPs324B corresponded to the neutral loss of H_2_O, (H_2_O + C_5_H_8_), and (H_2_O + C_5_H_8_ + H_2_O), respectively. PRO-TPs306B, PRO-TPs306C, and PRO-TPs306D were hydrolysates of PRO-TPs324. In addition, the former two might be hydrolyzed to form PRO-TPs238A, while PRO-TPs306D produced PRO-TPs220B with hydrolysis. As shown in Table S3 and Fig. 3, there formed four TPs with DIN photo-degradation. DIN parent ([M + H]^+^, *m*/*z* 326) went through cyclization directly with dehydrochlorination to produce DIN-TPs290C with the molecular formula C_15_H_16_ClN_3_. DIN was firstly isomerized, following the cyclization and hydroxyl oxidation to produce DIN-TPs290B, while generating DIN-TPs290A due to subsequent cyclization and dehydrochlorination. The structures of three isomers were corroborated by the characteristic fragment ions. DIN-TPs274 was derived from DIN-TPs 290A with hydroxyl loss. Compared with previous studies (Masis-Mora et al. 2019), there was a slight difference in these degradation products. As shown in Table S4 and Fig. 4, there were two major cyclization products due to the photo-degradation of TET. TET-TPs335 with the molecular formula of C_13_H_10_ClF_4_N_3_O came from the cyclized dehydrochlorination of TET parent, and it showed two characteristic ions (*m*/*z* 218.0 and 204.1) corresponding to the loss of C_2_HF_4_O and side chain, respectively. TET-TPs206 was generated from the side-chain oxidation of the TET parent molecule, simultaneous with the fragment loss of *m*/*z* 170.3 (HCl), *m*/*z* 164.1 (C_2_H_4_N), and *m*/*z* 102.0 (C_2_H_2_N_3_).

In short, three typical TFs underwent cyclization and dichlorination to form a series of nitrogenous heterocyclic compounds (NCHs), and these TPs exhibited higher polarity than parent itself. This result implied that TPs may be more toxic to aquatic organisms than parent to some extent, because they are easier to migrate to aquatic system by rain wash or soil leaching. Therefore, it is extremely necessary to focus on both TPs and parent compounds when a new treatment technology is developed.

### Optimal adsorption process

**Fig. 5:**
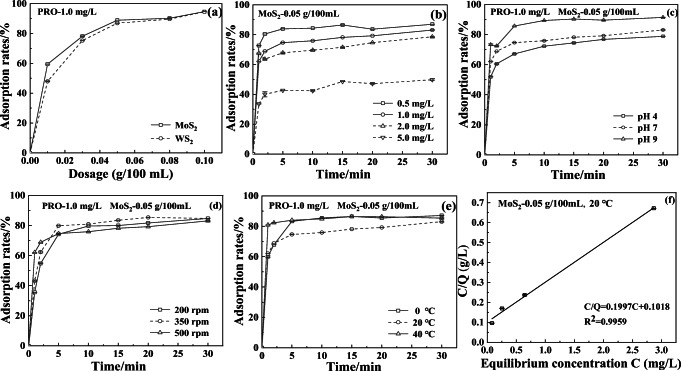
Optimization of the nano-MoS_2_ adsorption unit (PRO, 1.0 mg/L)

To determine the optimal dosages of MoS_2_ and WS_2_ on PRO aqueous solution (100 mL, *C*_0_ = 1.0 mg/L), the adsorption rates were investigated at the room temperature as shown in Fig. 5a. The results indicated that MoS_2_ exhibited a slightly better adsorption effect than WS_2_ in the range of < 0.08 g/100 mL aqueous solution, and no obvious increase in adsorption rate was observed when the dosage of MoS_2_ exceeded 0.05 g per 100 mL aqueous solution. Hence, the dosage of 0.05 g MoS_2_/100 mL solution (*C*_0_ = 1.0 mg/L) was used to optimize the parameters of adsorption process unit. The effects of initial concentration, pH value, agitation speed, and temperature on PRO removal rates were investigated. Firstly, PRO aqueous solution (*C*_0_ = 1.0 mg/L) was degraded for 30 min via the bench-scale photo-electrocatalytic reactor under the optimal conditions. Subsequently, the primary effluent (100 mL) was used to optimize the process parameters of secondary adsorption unit. As shown in Fig. 5b, the adsorption of MoS_2_ to PRO (0.5–5.0 mg/L) was almost saturated within 5 min for different initial concentrations, and PRO were removed from 5.0 to 2.87 mg/L, from 2.0 to 0.643 mg/L, from 1.00 to 0.254 mg/L, and from 0.5 to 0.081 mg/L, respectively. Obviously, the higher the initial PRO concentration was, the easier it was adsorbed by MoS_2_. This result might be interpreted by the separation factor (*R*_L_) of MoS_2_. The *R*_L_ values at different initial concentrations of PRO were calculated as the results of 0.5048 (0.5 mg/L), 0.3376 (1.0 mg/L), 0.2031 (2.0 mg/L), and 0.0925 (5.0 mg/L) on the basis of the slope (0.1997) and intercept (0.1018) of the isotherm adsorption equation (*R*^2^ = 0.9959) (Fig. 5f). There appeared a downward trend in *R*_L_ values with increasing initial concentrations of PRO, indicating that there was more conducive to adsorption of PRO. As shown in Fig. 5c, the adsorption rates of PRO (*C*_0_ = 1.0 mg/L, 0.05 g MoS_2_/100 mL) were 72.3% (pH 4), 75.9% (pH 7), and 89.4% (pH 9) within 10 min, respectively, indicating that the higher pH value was beneficial to the adsorption of MoS_2_ to PRO. The lower adsorption rate in acid medium might be attributed to the active site competition of MoS_2_ between PRO and hydrogen ion (Li et al. 2015). As can be seen from Fig. 5d, the stirring speed has little effect on the adsorption rate. The adsorption rate of PRO can reach 74.2% within 5 min even at 200 rpm. As depicted in Fig. 5e, although a low temperature was favorable for adsorption because it was an exothermic reaction, the saturated adsorption of MoS_2_ was almost unaffected by temperature in the range of 0–40 °C. In addition, the appropriate temperature increase was beneficial to adsorption equilibrium. In order to reduce consumption, the adsorption process was operated for 5 min under the optimized conditions (25 °C, 350 rpm, pH 9, 0.05 g MoS_2_/100 mL).

### Removal effects of TFs and TPs using the PECO-MoS_2_ technique

**Fig. 6:**
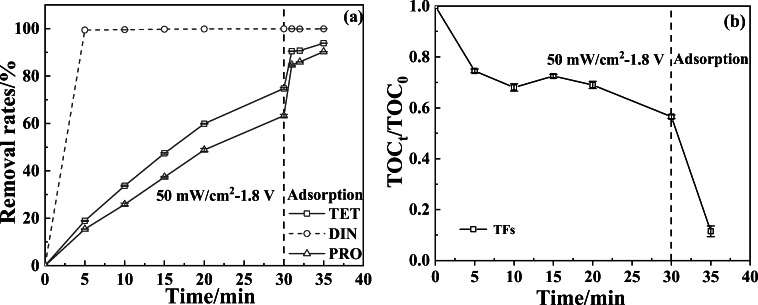
Removal rates (**a**) of TFs in actual surface waters, and TOC removal rates (**b**) (*C*_0_ = 1.0 mg/L) under optimal process

Under the optimized process conditions, the actual water sample–spiked TFs (1.0 mg/L, PRO, TET, and DIN) were treated to remove parent compounds and their TPs synchronously. The residues of PRO, TET, and DIN in primary effluent were 0.368 mg/L, 0.253 mg/L, and 0.0012 mg/L (close to detection limit), respectively, with the degradation rates of 63.3%, 74.7%, and 99.9%, respectively, after 30-min photo-electrocatalysis (Fig. 6a). After undergoing the adsorption of 5 min, the residues of PRO, TET, and DIN were declined to 0.0973 mg/L, 0.0617 mg/L, and 0.0012 mg/L, respectively, with the removal rates of 90.3%, 93.8%, and 99.9%, respectively. The mineralization degree of TFs in surface waters is shown in Fig. 6b. The ratio of TOC_t_ (at *t* min) and TOC_0_ (initial value) was declined to 0.57 undergoing 30-min photo-electrocatalysis. The TOC_15_ was slightly higher than TOC_10_, which might be attributed to the transformation of TFs to TPs. After the primary effluent was further treated for 5 min by MoS_2_ adsorption, the ratio of TOC/TOC_0_ was declined to 0.12, indicating that TFs and their TPs were removed by 88%. This result could be corroborated by the following fact. When the secondary effluent was concentrated 20 times by the QuECHERS-based method, no TPs in the enriched sample were detected in the full scan mode of LC-MS/MS. This result showed that these TPs can be absorbed completely by the MoS_2_ particle. This fact may be explained by the van der Waals force between triazole heterocycle and the S-edges of MoS_2_. This theory was supported by Salazar et al. (2019). From here, we see that the nano-MoS_2_ with a large specific surface area has been proved to be an excellent adsorbent to NHC-based pollutants, in view of similar nitrogen heterocycle structure.

### Potential cancer risk of effluent

As described in Table S5, the potential cancer risk coefficients induced by individual TFs were 1.13 × 10^−8^ (PRO), 7.78 × 10^−9^ (TET), and 3.69 × 10^−11^ (DIN), respectively, when TF aqueous solution was irradiated for 30 min by the reactor. In addition, the corresponding risk coefficients were further declined after undergoing 5-min adsorption by MoS_2_. Meanwhile, the total risk coefficients of three TFs were 1.91 × 10^−8^ (primary effluent) and 4.93 × 10^−9^ (secondary effluent), respectively. In view of maximum risk principle, the cancer risks of three TFs were far below the safe drinking standard (10^−6^) when the carcinogenic potency index (1.0) for each was used for calculation. Because no TPs were observed in secondary effluent after MoS_2_ adsorption, there was no need to feel anxious about the risk contribution of TPs.

## Conclusion

The typical TFs were photo-electrocatalytically degraded to a series of higher-polarity NHCs, and a tentative transformation pathway was proposed involving dehydrochlorination, cyclization, hydroxylation, etc. Although these TPs might pose potential aquatic toxicity, TFs and their TPs can be removed synchronously through the simple and effective tandem technique of PECO-MoS_2_ adsorption, especially in alkaline medium. The current study can provide theoretical basis for harmless treatment of TFs in the water environment. For better practical application, further research will be performed in the near future. For example, the magnetic MoS_2_-based adsorbent will be developed for facilitate recycling, simultaneous with regeneration method and recycling stability. Besides, the construction of doped photosensitive electrodes with higher oxygen evolution potential may be able to realize in situ synchronous mineralization of TFs and their TPs only by one-step photo-electrocatalysis.

## Supplementary information

ESM 1 (DOCX 929 kb)

## Data Availability

The datasets used and analyzed during the current study are available from the corresponding author on reasonable request.

## Abbreviations

CFD: Computational fluid dynamics
NCHs: Nitrogenous heterocyclic compounds
OER: Oxygen evolution reaction
PECO: Photo-electrocatalytic oxidation
PECR: Photo-electrocatalytic reactor
*R*_L_: Separation factor
SBSE: Stir bar sorptive extraction
TFs: Triazole fungicides
TPs: Transformation products
