# Supplementary material for: Degradation pathway of triazole fungicides and synchronous removal of transformation products via photo-electrocatalytic oxidation tandem MoS2 adsorption
Source: Environ Sci Pollut Res Int. 2021 Jan 2;28(13):16480–91. doi: 10.1007/s11356-020-12185-x (PMC7969552; doi:10.1007/s11356-020-12185-x)
Supplement: Supplementary file 1 — (DOCX 929 kb) [file 11356_2020_12185_MOESM1_ESM.docx]

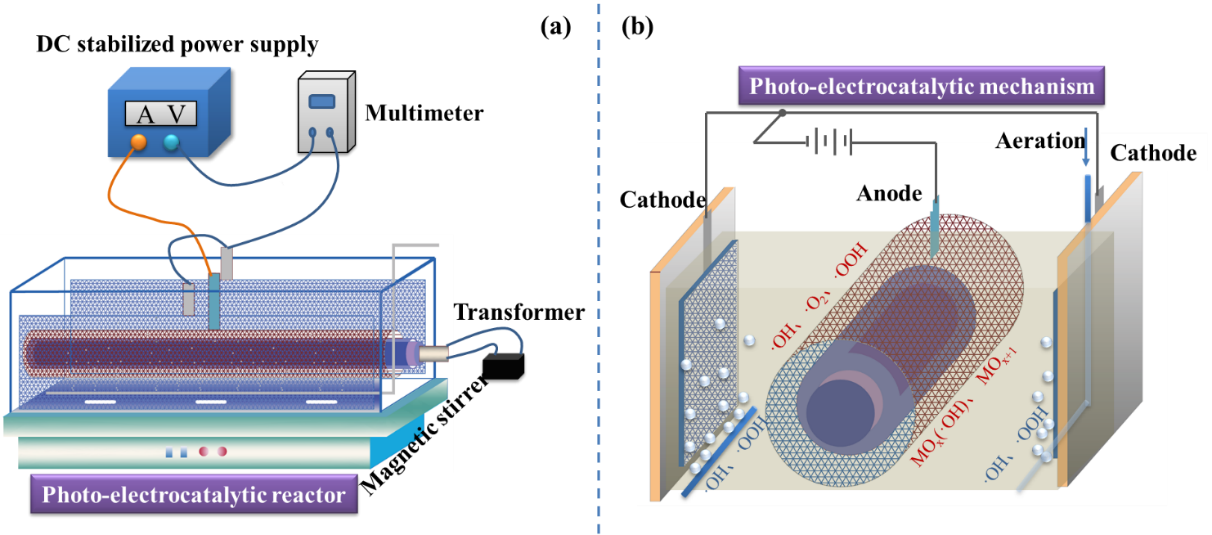


**Fig. S1. Structure of** **photo-electrocatalytic reactor (a) and mechanism (b).**

**Fig. S2. Effects of pH (a, b, c) and Fe(Ⅱ) (d, e, f) on degradation kinetics of DIN, TET, and PRO** **in aqueous solution (****0.05 mol/L Na_2_SO_4_).**

**Table S1. Theoretical redox potential of** **·OH/H_2_O at different pH values.**

| pH | E(·OH_aq_/H_2_O) |
| --- | --- |
| 4 | 2.354 V |
| 7 | 2.177 V |
| 9 | 2.059 V |

Note: Reaction: H_2_O→HO·+H^+^+e^-^, E(·OH_aq_/H_2_O)=E^θ^－0.059pH (E^θ^=2.59)

**Table S2. Data and structures of PRO-TPs using LC-MS/MS.**

| Compound | Structure | Molecular formula | Rt/min | m/z [M+H]  Experimental | Fragment | Product ion |
| --- | --- | --- | --- | --- | --- | --- |
| PRO-TPs  324A |  | C_15_H_18_ClN_3_O_3_ | 8.7 | 324.1 | 306.2  283.0  265.1  255.1  238.1 | [M + H –H_2_O]^+^  [M + H – C_3_H_5_]^+^  [M + H – C_3_H_5_– H_2_O]^+^  [M + H – C_3_H_5_– CO]^+^  [M + H – C_3_H_5_–H_2_O–C_2_H_3_]^+^ |
| PRO-TPs  324B |  | C_15_H_18_ClN_3_O_3_ | 3.8 | 324.2 | 306.1  238.0  220.0 | [M + H –H_2_O]^+^  [M + H –H_2_O–C_5_H_8_]^+^  [M + H –H_2_O–C_5_H_8_–H_2_O]^+^ |
| PRO-TPs  306A |  | C_15_H_16_ClN_3_O_2_ | 3.9 | 306.1 | 238.1  220.3 | [M + H – C_5_H_8_]^+^  [M + H – C_5_H_8_ – H_2_O]^+^ |
| PRO-TPs  306B |  | C_15_H_16_ClN_3_O_2_ | 2.4 | 306.2 | 237.2  220.0  169.2  151.2  123.0 | [M + H – C_5_H_9_]^+^  [M + H – C_5_H_9_ – OH]^+^  [M + H – C_5_H_9_ – OH – C_2_HN_3_ + O]^+^  [M + H – C_5_H_9_ – OH – C_2_HN_3_ + O – Cl + OH]^+^  [M + H – C_5_H_9_ – OH – C_2_HN_3_ + O – Cl + OH – CO ]^+^ |
| PRO-TPs  306C |  | C_15_H_16_ClN_3_O_2_ | 1.5 | 306.0 | 288.1  238.1  220.3  192.3 | [M + H – H_2_O]^+^  [M + H – C_5_H_8_]^+^  [M + H – C_5_H_8_ – H_2_O]^+^  [M + H – C_5_H_8_ – H_2_O – CO]^+^ |
| PRO-TPs  306D |  | C_15_H_16_ClN_3_O_2_ | 16.8 | 306.1 | 236.0  220.2  192.1 | [M + H – C_5_H_10_ ]^+^  [M + H – C_5_H_10_ – O]^+^  [M + H – C_5_H_10_ – O – CO]^+^ |
| PRO-TPs  238A |  | C_10_H_8_ClN_3_O_2_ | 2.0 | 238.1 | 220.1 | [M + H – H_2_O]^+^ |
| PRO-TPs  238B |  | C_10_H_5_Cl_2_N_3_ | 4.0 | 238.2 | 220.2 | [M + H – Cl + OH ]^+^ |
| PRO-TPs  220A |  | C_10_H_10_N_3_O_3_ | 2.0 | 220.1 | 192.2  176.8  164.3 | [M + H – CO]^+^  [M + H – C_2_H_2_ – OH]^+^  [M + H – CO – CH_2_N-]^+^ |
| PRO-TPs  220B |  | C_10_H_8_ClN_3_O_2_ | 4.0 | 220.2 | 191.2  176.8  164.3 | [M + H – CHO]^+^  [M + H – C_2_H_2_OH]^+^  [M + H – CHO – C_2_H_3_]^+^ |

**Table S3. Mass spectra data and structures of DIN-TPs using LC-MS/MS.**

| Compound | Structure | Molecular formula | Rt/min | m/z [M+H]  Experimental | Fragment | Product ion |
| --- | --- | --- | --- | --- | --- | --- |
| DIN-TPs  290A |  | C_15_H_16_ClN_3_O | 7.4 | 290.1 | 233.1  206.1 | [M + H –C_4_H_9_]^+^  [M + H – C_4_H_9_ – HCN]^+^ |
| DIN-TPs  290B |  | C_15_H_16_ClN_3_O | 1.4 | 290.2 | 233.1  204.1  177.8 | [M + H –C_4_H_9_]^+^  [M + H – C_4_H_9_ – CHO]^+^  [M + H – C_4_H_9_ – CHO – CN]^+^ |
| DIN-TPs  290C |  | C_15_H_16_ClN_3_O | 6.0 | 290.2 | 272.4  254.1  221.0  151.0 | [M + H –Cl + OH]^+^  [M + H – HCl]^+^  [M + H – triazole heterocycle]^+^  Loss of the left moiety (C_7_H_4_ClO) |
| DIN-TPs  274 |  | C_15_H_16_ClN_3_ | 2.0 | 274.4 | 256.2  230.3 | [M + H –Cl + OH]^+^  [M + H –Cl + OH – CN]^+^ |

**Table S4. Mass spectra data and structures of TET-TPs using LC-MS/MS.**

| Compound | Structure | Molecular formula | Rt/min | m/z [M+H]  Experimental | Fragment | Product ion |
| --- | --- | --- | --- | --- | --- | --- |
| TET-TPs  335 |  | C_13_H_10_CF_4_lN_3_O | 9.6 | 335.5 | 218.0  204.1 | [M + H –C_2_HF_4_O]^+^  [M + H –side chain]^+^ |
| TET-TPs  206 |  | C_10_H_8_ClN_3_ | 1.6 | 206.4 | 170.3  164.1  102.0 | [M + H – HCl]^+^  [M + H – C_2_H_4_N]^+^  [M + H – HCl – C_2_H_2_N_3_]^+^ |

**Table S5. Potential cancer risk coefficients of typical TFs in primary effluent and secondary effluent.**

| Compounds | Risk coefficients | |
| --- | --- | --- |
|  | Primary effluent  (30 min-PECO) | Secondary effluent  (10 min-adsorption) |
| PRO | 1.13×10^-8^ | 2.99×10^-9^ |
| TET | 7.78×10^-9^ | 1.90×10^-9^ |
| DIN | 3.69×10^-11^ | 3.69×10^-11^ |
| Sum of three TFs | 1.91×10^-8^ | 4.93×10^-9^ |

Note: the risk coefficients were calculated on basis of carcinogenic potency index (1.0) of TFs considering maximum risk.
